# Editorial: Protein phosphorylation and dephosphorylation in plant-microbe interactions

**DOI:** 10.3389/fpls.2022.1020772

**Published:** 2022-09-09

**Authors:** Jianbin Su, Yi-Ju Lu, Jens Staal, Agnieszka Ludwików

**Affiliations:** ^1^Division of Plant Sciences, University of Missouri, Columbia, MO, United States; ^2^Department of Plant, Soil and Microbial Sciences, Michigan State University, East Lansing, MI, United States; ^3^Institute of Biochemistry, National Chung Hsing University, Taichung, Taiwan; ^4^Department of Plant Pathology and Microbiology, National Taiwan University, Taipei, Taiwan; ^5^VIB Center for Inflammation Research, Ghent, Belgium; ^6^Department of Biomedical Molecular Biology, Ghent University, Ghent, Belgium; ^7^Laboratory of Biotechnology, Institute of Molecular Biology and Biotechnology, Faculty of Biology, Adam Mickiewicz University, Poznan, Poland

**Keywords:** protein phosphorylation, dephosphorylation, systemic acquired resistance (SAR), anthracnose, Agrobacterium, *Colletotrichum*, MKK4/MKK5, MPK3/MPK6

Protein phosphorylation and dephosphorylation play key roles in all types of host-microbe interactions, such as pattern-triggered immunity (PTI), effector-triggered immunity (ETI), induced resistance (IR), systemic acquired resistance (SAR) and symbiosis (Liang and Zhou, 2018; Albert et al., 2020; Li et al., 2020; Ngou et al., 2022; Sun and Zhang, 2022). Among them, PTI-related phosphorylation events have been extensively studied in the past 30 years (Albert et al., 2020; Ngou et al., 2022). During PTI, plasma membrane-localized receptor-like kinases (RLKs) or receptor-like proteins (RLPs) sensing the presence of conserved pathogen-associated molecular patterns (PAMPs). Perception of PAMPs by RLKs/RLPs triggers the activation of receptor-like cytoplasmic kinases (RLCKs), a subset of RLKs lacking extracellular ligand-binding domain. Activated RLCK then activates a series of subsequent signaling events, including mitogen-activated protein kinase (MAPK) activation, reactive oxygen species (ROS) burst, calcium spiking and cytoskeleton remodeling. In the case of flagellin sensing, BIK1, a RLCK downstream of FLS2, was shown to phosphorylate NADPH oxidase RBOHD and two calmodulin-gated calcium channel CNGC2 and CNGC4, which activate ROS burst and cytoplasmic calcium spiking, respectively in Arabidopsis (Liang and Zhou, 2018; Tian et al., 2019). As a second messenger, calcium spiking activates further phosphorylation signaling events to fine-tune signaling output. Arabidopsis calcium-dependent protein kinases (CPKs), CPK1-6 positively, while CPK28 negatively regulate PTI (Yip Delormel and Boudsocq, 2019). The amplitude and duration of kinase activations during PTI are also fine-regulated by phosphatase-mediated dephosphorylation (Li et al., 2020). Though detailed molecular phosphorylation mechanisms are not extensively elucidated in ETI, IR and SAR, accumulating data suggest that their phosphorylation signaling components are largely overlapping with those found in PTI.

This Research Topic provides an update on protein kinases in host-microbe interactions including a review summarizing protein kinase signaling pathways in plant-*Colletotrichum* interaction (Jiang et al.); two phosphoproteomics studies, one focusing on dynamic changes of phosphoproteins in SAR (Zhou et al.), the other identified novel putative targets of an atypical kinase ILK1 (Brauer et al.); two classical genetic studies, one demonstrates the requirement of Arabidopsis MKK4/MKK5-MPK3/MPK6 signaling module in suppressing Agrobacterium-mediated gene transformation (Liu et al.); the other characterized a fungal calcium/calmodulin-dependent protein kinase (CAMK) (Pan et al.).

*Colletotrichum spp*. is a causal agent of anthracnose disease on crops, trees, and vegetables. In the review paper, the authors summarized the current knowledge on protein kinase pathways from both the host and pathogen perspectives with the emphasis on non-model plants (Jiang et al.). In addition to the requirement of cyclic adenosine monophosphate (cAMP) – protein kinase A (PKA) signaling, MAPK signaling,_morphogenesis-related NDR kinase pathway (MOR) pathway and two-component phosphorelay system (TCSs) for *Colletotrichum* virulence (Jiang et al.). Pan et al. found that CAMK CgSgt4 is also required for full virulence of *Colletotrichum gloeosporioides*, a fungal pathogen causing anthracnose disease in many tropical fruits. We believe these fundamental studies on fungal kinases would greatly facilitate the development of kinase inhibitor-based fungicide.

Phosphoproteomics has been successfully applied to studying dynamic phosphorylation events during PTI and ETI (Benschop et al., 2007; Nühse et al., 2007; Kadota et al., 2019). To date, phosphoproteome changes during SAR have not been explored. Through quantitative phosphoproteomics, Zhou et al. identified 859 significantly changed phosphoproteins in systemic leaves 48h after *Psm ES4326* treatment, at a time point when SAR has been successfully established. In future, it would still be interesting to explore dynamic changes of phosphoproteome during the establishment of SAR with short time points. Another proteomic study in this Research Topic screened putative targets and interacting proteins of ILK1, an atypical kinase belonging to the ILK family of Raf-like MAP3K (Brauer et al.). Though ILK1 is classified as a pseudo-kinase, it showed kinase activity *in vitro*, with unusual preference for Mn2^+^ as cofactor (Brauer et al., 2016). ILK1 activity is essential for flg22-, elf18- and pep1-induced root growth inhibition (Brauer et al., 2016). Recently, ILK5 was shown to be a true MAP3K function upstream of MKK5-MPK3/MPK6 in extracellular perception (Kim et al., 2022). The identification of MEEK1 and MEKK3 as potential ILK1 interactors encourages future work to test whether ILK1 functions as MAP3K or MAP4K in PTI.

In plants, the MKK4/MKK5-MPK3/MPK6 is the key module functioning downstream of many MAP3Ks in both development and immunity (Jagodzik et al., 2018; Sun and Zhang, 2022). Previous work suggested MPK3 promote Agrobacterium-mediated gene transformation by phosphorylating of VIP1 (Pitzschke et al., 2009). However, an original article in this Research Topic suggests that MKK5/MKK5-MPK3/MPK6 negatively regulates Agrobacterium-mediated gene transformation with classical genetic studies (Liu et al.). Enhanced transformation was observed in both *mkk4 mkk5* double and *mpk3 mpk6* mutants (Liu et al.). Accordingly, inducible activation of MPK3/MPK6 by MKK4 or MKK5 suppressed Agrobacterium-mediated gene transformation (Liu et al.). It worths noting that MPK3/MPK6 activation is transient during Agrobacterium treatment (Liu et al.), but is long-lasting when induced by inducible expression of upstream constitutive MEKs (Su et al., 2018). Though *mkk4 mkk5* and *mpk3 mpk6* mutants showed an increased transformation ratio, the possibility that MPK3 promotes transformation by phosphorylating VIP1 could still not be excluded. Thus, the role of MKK4/MKK5-MPK3/MPK6 in Agrobacterium-mediated gene transformation is remain to be examined further.

Altogether, these articles in this Research Topic highlight the importance and complexity of protein phosphorylation in different types of plant-microbe interactions. With rapid improvements in phosphoproteomics and precision gene editing technologies, comprehensive phosphorylation regulatory networks and mechanisms will be revealed.

## Author contributions

JSu prepared the first draft of this editorial. Y-JL, JSt, and AL revised the editorial. All authors approved it for publication.

## Funding

JSu is supported by National Science Foundation grant IOS-1456181. Y-JL is supported by funding from the National Institutes of General Medical Sciences (1R01GM125743) and internal fundings from National Chung-Hsing University and National Taiwan University. JSt is supported by Fund for Scientific Research-Flanders (FWO) grant (G021119N) and a Ghent University Concerted actions (GOA) grant (BOF19-GOA-004). AL is supported by the National Science Centre grants: 2014/15/B/NZ3/00358, 2016/22/E/NZ3/00345, and 2021/41/B/NZ3/00711.

## Conflict of interest

The authors declare that the research was conducted in the absence of any commercial or financial relationships that could be construed as a potential conflict of interest.

## Publisher's note

All claims expressed in this article are solely those of the authors and do not necessarily represent those of their affiliated organizations, or those of the publisher, the editors and the reviewers. Any product that may be evaluated in this article, or claim that may be made by its manufacturer, is not guaranteed or endorsed by the publisher.
